# Mindful Eating Proficiency and Healthy Eating Literacy among Japanese Mothers: Associations with Their Own and Their Children’s Eating Behavior

**DOI:** 10.3390/nu13124439

**Published:** 2021-12-11

**Authors:** Taro Nakamura, Rie Akamatsu, Nobuo Yoshiike

**Affiliations:** 1 Graduate School of Health Sciences, Aomori University of Health and Welfare, 58-1 Mase, Hamadate, Aomori-Shi, Aomori 030-8505, Japan; n_yoshiike@auhw.ac.jp; 2 Natural Science Division, Faculty of Core Research, Ochanomizu University, 2-1-1 Otsuka, Bunkyo-ku, Tokyo 112-8610, Japan; akamatsu.rie@ocha.ac.jp

**Keywords:** mindful eating, healthy eating literacy, mothers, children, eating behavior, Japan

## Abstract

Mindfulness is a process of focusing one’s attention on the present moment. Applying this concept to eating (i.e., mindful eating (ME)) is associated with regulated eating behaviors, particularly in people with obesity and who are overweight. Sustaining healthy eating habits requires both healthy eating literacy (HEL) and proficiency in ME. However, ME proficiency in Japanese people has not been sufficiently investigated. In this paper, we conduct a survey of mothers with 4- to 5-year-old children in Aomori City, Japan, to investigate their ME proficiency and HEL level and eating behavior and self-reported body mass index in both mothers and their children from August to September 2019. This study is the first to describe ME proficiency in Japanese mothers. The study sample includes 128 participants from 18 nursery schools. ME proficiency in mothers was positively correlated with both their own and their children’s eating behaviors, thereby suggesting a potential relationship, while strong relationships were not observed between the HEL level and eating behaviors of mothers and children. Improving ME skills, rather than HEL, may be an effective way to sustain healthier eating behaviors in mothers and their children. The level of evidence was Level V: Opinions of respected authorities based on descriptive studies, narrative reviews, clinical experience, or reports of expert committees.

## 1. Introduction

Mindfulness is a process of focusing one’s attention on the present moment [1,2,3]. The application of mindfulness to eating has developed into a personal approach to improving eating behaviors, which is referred to as mindful eating (ME) [4].

Regarding ME and its practice, previous research has suggested that ME includes the awareness of internal and external cues that influence the desire to eat, food choices, the amount eaten, and the manner in which food is eaten [5]. Mindful eating also includes making conscious choices and learning to be more aware of cues that indicate fullness [6]. Paying attention to these two factors has been shown to lead to healthier eating [7]. Furthermore, ME includes being aware of hunger and satiety, eliminating distractions, knowing the consequences of eating inattentively, choosing appealing and nutritious food, and judging how much to eat [8].

Studies have demonstrated ME’s effectiveness in treating eating disorders in obese women [9]. ME may also effectively manage binge eating [6,10,11,12,13,14], emotional eating [15,16], external eating [15,17], cravings [18,19], hunger awareness [20], food intake [7,20,21,22], and food choice [22,23]. Numerous studies have also demonstrated that ME may be associated with weight loss and may be a practical approach toward weight loss [6,10,12,16,17,19,20,21,22].

Healthy eating literacy (HEL) is another approach to manage eating behavior. The HEL scale is based on a modified health literacy (HL) developed by Ishikawa et al. [24], which consists of sub-concepts of interactive and critical health literacy. In contrast to ME, which is characterized by dietary awareness and mindfulness, literacy is the ability to use cognitive skills to understand, organize, and apply a descriptive system to enact change. HEL has been associated with behavioral transformation, including practicing healthier eating [25]. It has been also shown that mothers with high HEL actually prepared a healthy diet at home to ensure a nutritionally balanced diet [26]. The National Nutrition Survey on Preschool Children provides fundamental information for preschooler’s diet and lifestyle [27]. According to another study [28], “fruits, vegetables and high-protein foods” patterns and “confectionaries and sweetened beverages” patterns were identified among infants aged 16–24 months. However, there are not many reports of dietary patterns and behavior in families with preschoolers in Japan. Moreover, though learning and practicing healthier eating requires improvement in both HEL and ME proficiency, no studies have been published on whether ME or HEL has a greater effect on eating behavior or on how they may interact to influence eating behavior.

Furthermore, it is just as important to pass down healthy eating habits to children. Although many believe that a mother’s eating behavior is passed onto their children [29,30], no studies have demonstrated the possible effects of a mother’s ME proficiency on their children’s eating behaviors. Therefore, the purpose of this study is to investigate (1) how proficient Japanese mothers are at practicing ME; (2) how their ME proficiency and HEL level relate to their eating behaviors; and (3) how those factors may relate to their children’s eating behaviors.

## 2. Materials and Methods

### 2.1. Design and Participants

Aomori Prefecture, with more than 1.2 million people, is located at the north end of Honshu, in the main island of Japan. It is one of the cities with dietary problems, such as excessive salt intake and low vegetable intake [31]. Moreover, it has prioritized nutrition issues with a focus on the control of overweight people and obesity in their health promotion plan established in 2000. For these reasons, Aomori Prefecture was selected as the target city. A total of 20 nursery schools from 54 schools in Aomori City were randomly selected, of which 18 agreed to participate. From August through September 2019, all mothers with 4- to 5-year-old children attending one of those nursery schools received an anonymous self-administered questionnaire to be completed at home and collected at their school. There were no other criteria for selecting the participants.

### 2.2. Data Collection

The questionnaires were completed at home and collected through the nursery schools. It comprised 77 items: 8 demographic items, a Japanese version of the 20-item Italian Mindful Eating Questionnaire (MEQ) [32], the 5-item HEL [25], and two 22-item Eating Behavior Scales (EBS), 1 for the mother to complete about herself and the other regarding her child. Informed consent was obtained from all participants after providing them with information about the study’s purpose and method, and assuring that participation was voluntary, with their identity being protected.

### 2.3. Japanese Version of the 20-Item Italian MEQ

To assess ME proficiency, we used a Japanese version of the 20-item Italian MEQ, for which the validity and reliability had been confirmed [32]. The 20 items measure 4 factors—disinhibition, awareness, external cues, and emotional response—using a 4-point scale. After creating a Japanese version, it was back translated into English, and the equivalence of the original version and the back translated version were examined by the authors. We confirmed that it matched the concept that Clementi et al. had proposed [32]. The higher the ME scores, the more proficient their mindful eating.

### 2.4. The Healthy Eating Literacy Scale

The HEL scale, whose validity and reliability were confirmed [25], was used to measure HEL. It uses a five-point scale and includes five items, namely, three measuring interactive literacy and two measuring critical literacy in food information.

### 2.5. The Eating Behavior Scale

Finally, two EBS were used to assess eating behavior healthiness for the mother and her child, both to be completed by the mother. The scales’ validity and reliability were confirmed [33]. The EBS comprises 22 items measuring 4 factors (i.e., binge eating, dietary balance, eating rhythm, and manner of eating) and is scored on a 5-point scale. The higher the EBS, the healthier their eating behavior.

### 2.6. Demographic Items

Respondents were asked to indicate their age, highest level of education, employment status, household income, marital status, sex of child, number of children, and the mother’s self-reported height and weight to calculate body mass index.

### 2.7. Sample Size Calculations and Statistical Analyses

Aomori City’s nursery schools have approximately 700 children aged 4–5 years, and, as such, a sample size of approximately 80 would be representative of Aomori City (α-error 0.05, power (1–0.10)). Assuming a 60% response rate and a 50% valid response rate, we determined that 270 questionnaires needed to be distributed.

Descriptive statistics for the sample were reported using percentages for the categorical variable and means and standard deviations for the continuous variables. After confirming that ME proficiency and HEL scores were normally distributed using the Kolmogorov–Smirnov test, forced-entry multiple regression was performed with ME proficiency and HEL as the response variables and participant characteristics as the explanatory variables. The factors presented by the nominal scale in this paper were converted to dummy variables. Multiple regression was similarly performed with mothers’ EBS as response variables and ME, HEL, and participant characteristics as explanatory variables. Pearson’s correlation coefficient (r) was used to investigate correlations between mothers’ EBS scores and their ME proficiency and HEL scores, between mother and child EBS scores, and between EBS scores and BMI for the mothers. The Cronbach’s alpha coefficient was used as the measure of the reliability of the ME questionnaire. Cronbach’s alpha coefficients of the overall scale and 4 subscales of the ME questionnaire were 0.740, 0.733, 0.726, 0.734, and 0.726, respectively. Using the median scores for ME proficiency and HEL, the sample was divided into four ME–HEL proficiency groups: Group A (ME ≥ 2.80, HEL ≥ 3.60), Group B (ME ≥ 2.80, HEL < 3.60), Group C (ME < 2.80, HEL ≥ 3.60), and Group D (ME < 2.80, HEL < 3.60). The chi-squared test was used to identify significant between-group differences in categorical variables representing participant characteristics. For the continuous variables (ME, HEL, EBS, and BMI), a two-way analysis of variance (ANOVA) was performed followed by post-hoc multiple comparisons by using Tukey’s Test. R ver. 3.5.2 was used for the statistical analysis with the significance level set at *p* < 0.05 (2-tailed test).

Of the 270 questionnaires distributed, 177 were returned (response rate = 65.6%). Of these, four participants did not include informed consent. After eliminating surveys with missing data, the analysis sample included 128 participants.

## 3. Results

### 3.1. Participant Characteristics

Participant characteristics are shown in (Table 1). For mothers, the mean scores were: ME, 2.85 ± 0.31; HEL, 3.60 ± 0.62; and EBS score, 3.35 ± 0.55. The mothers’ mean BMI was 21.50 ± 3.39 kg/m^2^.

### 3.2. Estimating the Effects of Participant Characteristics on ME, HEL, and EBS

Forced-entry multiple regression was performed on the entire sample by using ME and HEL as response variables and demographic items, namely age, education level, employment status, household income, marital status, sex of child, and the total number of children in the family as explanatory variables. The results showed that age, highest level of education, employment status, household income, marital status, sex of child, and number of children were not very important predictors of ME or HEL (Table 2 and Table 3).

### 3.3. Mothers’ EBS by ME–HEL Group

**Figure 1:**
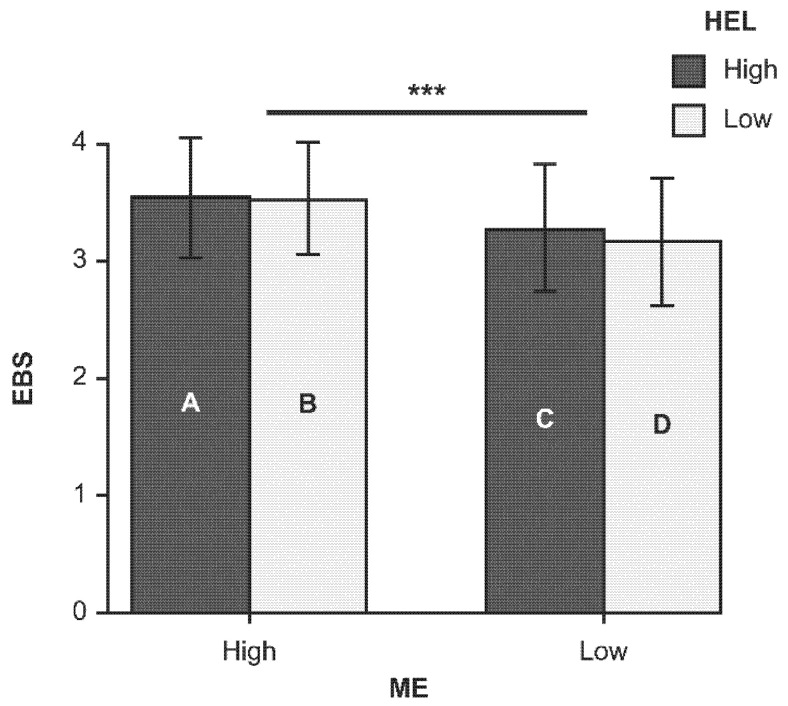
Effects of ME and HEL on EBS. Note: Capital letters A, B, C, and D indicate group names. Group A (ME ≥ 2.80, HEL ≥ 3.60), Group B (ME ≥ 2.80, HEL < 3.60), Group C (ME < 2.80, HEL ≥ 3.60), and Group D (ME < 2.80, HEL < 3.60). Data are shown as mean ± S.D. *** *p* < 0.001.

A positive correlation was found between ME proficiency and mothers’ EBS (r = 0.43, *p* < 0.001), whereas the correlation between mothers’ EBS and HEL was weak (r = 0.17, *p* < 0.05). Given that the purpose of the study was to understand the effect of different levels of ME proficiency and HEL in Japanese mothers on their own and their children’s EBS, we performed an ME–HEL category analysis. Using the median values for ME and HEL scores (2.80 and 3.60, respectively), the sample was divided into four groups: Group A (ME ≥ 2.80, HEL 3.60), Group B (ME ≥ 2.80, HEL < 3.60), Group C (ME < 2.80, HEL ≥ 3.60), and Group D (ME < 2.80, HEL < 3.60). Chi-squared test results showed no significant between-group differences in participant characteristics (Table 4). A two-way ANOVA test revealed no significant interaction between ME and HEL on EBS, but very significant main effects for ME (Df = 1, F = 11.5135, *p* < 0.001), thereby indicating that these factors acted independently (Figure 1 and Table 5). The results showed that ME had a significantly greater effect on EBS than HEL in mothers. For BMI in mothers, no significant between-group differences were found.

### 3.4. EBS Scores and BMI

A significant correlation was observed between the mothers’ and child’s EBS scores (r = 0.55, *p* < 0.001), but not between EBS scores and BMI in the mothers (r = −0.02, *p* = 0.783).

## 4. Discussion

To our knowledge, this study is the first to measure ME proficiency in Japanese mothers. Mothers’ EBS scores positively correlated with ME proficiency, whereas no strong correlation emerged between mothers’ EBS scores and HEL. Mother and child EBS scores were significantly related. These results suggest that maintaining healthy eating behaviors in both mothers and their children may be associated with the mother’s proficiency at practicing ME, in addition to social and environmental factors.

From the Cronbach’s alpha coefficient of each ME questionnaire item, no major problem in the ME scale’s reliability was observed. The mean ME proficiency score in the present study (i.e., 2.85 ± 0.31) was similar to those found in a previous study, despite differences in sex, age, and race [34]. This may indicate that ME proficiency in Japanese mothers is not low.

The mean HEL score in the present study (i.e., 3.60 ± 0.62) largely matched the results of prior studies [25,35,36]. Although literacy on a healthy diet was generally associated with dietary behavior, no strong relationship between HEL and EBS was found. This may be because healthy eating behavior in mothers is more influenced by other factors, such as social and environmental factors, than their literacy [37].

In the analyses of the ME–HEL group, no significant between-group differences were found in participant characteristics (Table 4). The results of the two-way ANOVA suggested that the mothers’ EBS score was more affected by ME than by HEL (Figure 1 and Table 5). It may suggest that focusing on the diet one is eating is more important than increasing dietary literacy to improve eating behavior. These results also indicate that ME proficiency can be improved posteriorly, which may lead to an improvement in EBS [38].

A significant positive correlation was found between mothers’ and their child’s EBS scores, which is consistent with the findings of another study, for example [39]. A child’s eating behavior is also associated with their current health status, along with their physical and emotional development [40]. Establishing a healthy eating behavior in childhood is crucial because it can affect their eating habits and thus their health in adulthood. No relationship was found between eating behaviors and BMI in the mothers. The number of overweight subjects (BMI > 25.0) was lower (16 (12.5%)), which is considered to be one of the reasons. In addition, this may be because many factors outside of eating behavior can affect weight, such as exercise and sleep, as well as drinking and smoking in adults [41].

This study has two strengths. First, the ME proficiency of Japanese mothers was revealed for the first time, and thus, it is expected to be the basis for future research on the development of mindful eating in Japan. Second, it investigated how the mother’s ME proficiency and HEL level were associated with their dietary behavior. Because the current primary method was to improve eating behavior by increasing HEL in nutrition education, adding the ME concept to dietary education is expected to lead to proper eating behaviors of mothers and children [26]. This study also had three important limitations, the first of which is the cross-sectional design. Despite finding a potential relationship between ME proficiency and eating behavior, the two factors were not measured independently because a questionnaire was used; therefore, a cause-and-effect relationship could not be determined, and an intervention study is necessary to investigate causality. The second limitation is the validity of using the ME scale. Clementi et al. developed the ME scale with Italian adults in mind [32]. We adopted the scale to investigate Japanese mothers’ ME proficiency; however, it may not accurately reflect Japanese culture and customs despite the careful confirmation by the authors. The final limitation is that the sample size was not large enough to find any participant characteristic affecting ME, HEL, and EBS, although it is generally believed that age, household income, educational background, among others, have an effect. To reverify these results, a multivariate analysis was performed by adding samples, including missing data, but the results were unclear. It may be necessary to increase the sample size or change the question items to verify them.

The simultaneous investigation of the effects of ME and HEL on eating behavior suggested that ME had more of an effect than HEL. Based on this result, we speculated that improving ME skills may be an effective approach to sustain healthier eating behaviors in mothers and their children. Because this was the first time that the measurement of ME proficiency was used in a study of Japanese people, the study provides a basis for future research in Japan.

## 5. Conclusions

Using a sample of mothers with 4- and 5-year-olds attending nursery schools in Aomori City, Japan, this study examined ME proficiency and HEL as well as eating behavior and BMI in the mothers. To the best of our knowledge, this was the first time ME was studied in Japanese mothers. In these mothers, ME proficiency appeared to influence eating behavior more than HEL and their eating behaviors appeared to influence their children’s eating behaviors. Therefore, to sustain healthier eating behaviors in Japanese mothers and their children, it may be effective to help mothers eat more mindfully in addition to improving their HEL.

## Figures and Tables

**Table 1:** Participant characteristics.

Age	N (%) ^a^
20s	15 (11.7)
30s	82 (64.1)
40s	31 (24.2)
Highest level of education	
Middle school/High school	49 (38.3)
Junior college/Vocational school	52 (40.6)
University/Postgraduate degree	27 (21.1)
Employment status	
Full-time	86 (67.2)
Part-time	40 (31.3)
Unemployed	2 (1.6)
Household income (yen/year)	
<2 million	15 (11.7)
2–4 million	48 (37.5)
4–6 million	38 (29.7)
6–8 million	11 (8.6)
>8 million	16 (12.5)
Marital status	
Married	108 (84.4)
Widowed/Divorced	18 (14.1)
Never married	2 (1.6)
Sex of the child in the study	
Male	72 (56.3)
Female	56 (43.8)
Number of children	
1	24 (18.8)
2	73 (57.0)
3	23 (18.0)
4	7 (5.5)
5	1 (0.8)

^a^ Values are expressed as total N (%). Only includes participants with no missing data.

**Table 2:** Estimated Effects of Participant Characteristics on Mindful Eating.

	Independent Variable	Partial Regression Coefficient	*p*-Value	95% CI ^a^	
				Lower Limit	Upper Limit
Age ^b^	30s	0.169	0.152	−0.063	0.401
	40s	0.116	0.360	−0.134	0.367
Highest level of education ^c^	Junior college/Vocational school	−0.044	0.549	−0.187	0.100
	University/Postgraduate degree	0.001	0.992	−0.164	0.166
Employment status ^d^	Part-time	−0.015	0.823	−0.144	0.115
	Unemployed	0.105	0.644	−0.344	0.554
Household income ^e^	2–4 million yen	0.163	0.139	−0.054	0.379
	4–6 million yen	0.156	0.220	−0.095	0.406
	6–8 million yen	0.217	0.123	−0.060	0.495
	>8 million yen	0.297	0.039	0.015	0.579
Marital status ^f^	Widowed/Divorced	0.248	0.030	0.024	0.472
	Never married	−0.087	0.740	−0.608	0.434
Sex of child ^g^	Female	−0.035	0.558	−0.151	0.082
Number of children ^h^ (including the child in the study)	2	−0.099	0.225	−0.259	0.062
	3	−0.184	0.063	−0.379	0.010
	4	0.079	0.573	−0.198	0.355
	5	−0.342	0.287	−0.976	0.292

^a^ CI: confidence interval. Group of reference: ^b^ 20s, ^c^ Middle school/High school, ^d^ Full-time, ^e^ <2 million yen, ^f^ Married, ^g^ Male, and ^h^ 1.

**Table 3:** Estimated effects of participant characteristics on healthy eating literacy.

	Independent Variable	Partial Regression Coefficient	*p*-Value	95% CI ^a^	
				Lower Limit	Upper Limit
Age ^b^	30s	−0.318	0.181	−0.787	0.151
	40s	−0.365	0.156	−0.872	0.141
Highest level of education ^c^	Junior college/Vocational school	−0.134	0.361	−0.425	0.156
	University/Postgraduate degree	0.010	0.952	−0.323	0.344
Employment status ^d^	Part-time	0.159	0.230	−0.102	0.420
	Unemployed	−0.037	0.935	−0.944	0.869
Household income ^e^	2–4 million yen	−0.126	0.568	−0.564	0.311
	4–6 million yen	−0.219	0.392	−0.725	0.287
	6–8 million yen	0.186	0.513	−0.374	0.746
	>8 million yen	0.164	0.570	−0.406	0.734
Marital status ^f^	Widowed/Divorced	−0.113	0.622	−0.565	0.339
	Never married	−0.994	0.064	−2.045	0.058
Sex of child ^g^	Female	0.135	0.259	−0.101	0.370
Number of children ^h^ (including the child in the study)	2	−0.226	0.169	−0.550	0.098
	3	−0.172	0.387	−0.566	0.221
	4	−0.429	0.131	−0.986	0.129
	5	−0.228	0.725	−1.508	1.052

^a^ CI: confidence interval. Group of reference: ^b^ 20s, ^c^ Middle school/High school, ^d^ Full-time, ^e^ <2 million yen, ^f^ Married, ^g^ Male, and ^h^ 1.

**Table 4:** Number of participants by characteristic and mindful eating–healthy eating literacy (ME–HEL) group.

N (%) ^a^	Group A	Group B	Group C	Group D	*p*-Value ^b^
Age					0.788
20s	8 (17.8)	2 (6.7)	3 (12.0)	2 (7.1)	
30s	29 (64.4)	19 (63.3)	15 (60.0)	19 (67.9)	
40s	8 (17.8)	9 (30.0)	7 (28.0)	7 (25.0)	
Education level					0.488
Middle school/High school	20 (44.4)	10 (33.3)	9 (36.0)	10 (35.7)	
Junior college/Vocational school	14 (31.1)	16 (53.3)	9 (36.0)	13 (46.4)	
University/Postgraduate degree	11 (24.4)	4 (13.3)	7 (28.0)	5 (17.9)	
Employment status					0.950
Full-time	30 (66.7)	19 (63.3)	17 (68.0)	20 (71.4)	
Part-time	14 (31.1)	10 (33.3)	8 (32.0)	8 (28.6)	
Unemployed	1 (2.2)	1 (3.3)	0 (0.0)	0 (0.0)	
Household income					0.269
<2 million yen	6 (13.3)	2 (6.7)	3 (12.0)	4 (14.3)	
2–4 million yen	20 (44.4)	13 (43.3)	9 (36.0)	6 (21.4)	
4–6 million yen	7 (15.6)	8 (26.7)	7 (28.0)	16 (57.1)	
6–8 million yen	5 (11.1)	2 (6.7)	4 (16.0)	0 (0.0)	
>8 million yen	7 (15.6)	5 (16.7)	2 (8.0)	2 (7.1)	
Marital status					0.303
Married	34 (75.6)	25 (83.3)	23 (92.0)	26 (92.9)	
Widowed/Divorced	10 (22.2)	5 (16.7)	2 (8.0)	1 (3.6)	
Never married	1 (2.2)	0 (0.0)	0 (0.0)	1 (3.6)	
Sex of the child in the study					0.794
Male	25 (55.6)	17 (56.7)	12 (48.0)	18 (64.3)	
Female	20 (44.4)	13 (43.3)	13 (52.0)	10 (35.7)	
Total number of children					0.253
1	13 (28.9)	4 (13.3)	3 (12.0)	4 (14.3)	
2	21 (46.7)	20 (66.7)	17 (68.0)	15 (53.6)	
3	8 (17.8)	3 (10.0)	4 (16.0)	8 (28.6)	
4	3 (6.7)	3 (10.0)	0 (0.0)	1 (3.6)	
5	0 (0.0)	0 (0.0)	1 (4.0)	0 (0.0)	

^a^ Values are expressed by group. ^b^ *p*-value obtained in Chi-square test.

**Table 5:** Two-way ANOVA Test for the EBS by ME and HEL in Mothers.

Source	SS	df	F	*p*-Value
(Intercept)	1391.75	1	5135.4788	<0.001
ME	3.12	1	11.5135	<0.001
HEL	0.13	1	0.4624	0.498
ME x HEL	0.08	1	0.2952	0.588
Residuals	33.60	124		

## Data Availability

Not applicable.
